# COVID-19 Vaccine Hesitancy Among Older Adolescents and Young Adults: A National Cross-Sectional Study in China

**DOI:** 10.3389/fpubh.2022.877668

**Published:** 2022-05-12

**Authors:** Panpan Zhang, Yan Li, Huanchun Wang, Liyan Luo, Ping Wang, Huimin Wang, Qing Li, Zejing Meng, Hui Yang, Yuanhong Liu, Shiyue Zhou, Nan Li, Shengnan Zhang, Jianzhong Bi, Jiewen Zhang, Xiaolei Zheng

**Affiliations:** ^1^Department of Neurology, People's Hospital of Zhengzhou University, Zhengzhou, China; ^2^Department of Neurology, Puyang People's Hospital, Puyang, China; ^3^Department of Neurology, Yishui County People's Hospital, Linyi, China; ^4^Department of Basic Medicine Teaching, Taishan Vocational College of Nursing, Taian, China; ^5^Department of Epidemiology, School of Public Health, Cheeloo College of Medicine, Shandong University, Jinan, China; ^6^Department of Neurology, The Second Hospital, Cheeloo College of Medicine, Shandong University, Jinan, China; ^7^Department of Oral and Maxillofacial Surgery, Puyang People's Hospital, Puyang, China; ^8^Department of Public Health, School of Public Health, Lanzhou University, Lanzhou, China; ^9^Department of Neurology, Sishui County People's Hospital, Jining, China

**Keywords:** COVID-19, vaccine hesitancy, older adolescents, young adults, PSI-Y

## Abstract

**Background:**

With promotion of COVID-19 vaccinations, there has been a corresponding vaccine hesitancy, of which older adolescents and young adults represent groups of particular concern. In this report, we investigated the prevalence and reasons for vaccine hesitancy, as well as potential risk factors, within older adolescents and young adults in China.

**Methods:**

To assess these issues, an online survey was administered over the period from March 14 to April 15, 2021. Older adolescents (16–17 years old) and young adults (18–21 years old) were recruited nationwide from Wechat groups and results from a total of 2,414 respondents were analyzed. Socio-demographic variables, vaccine hesitancy, psychological distress, abnormal illness behavior, global well-being and social support were analyzed in this report.

**Results:**

Compared to young adults (*n* = 1,405), older adolescents (*n* = 1,009) showed higher prevalence rates of COVID-19 vaccine hesitancy (16.5 vs. 7.9%, *p* < 0.001). History of physical diseases (*p* = 0.007) and abnormal illness behavior (*p* = 0.001) were risk factors for vaccine hesitancy among older adolescents, while only a good self-reported health status (*p* = 0.048) was a risk factor for young adults. Concerns over COVID-19 vaccine side effects (67.1%) and beliefs of invulnerability regarding infection risk (41.9%) were the most prevalent reasons for vaccine hesitancy. Providing evidence on the vaccine reduction of COVID-19 infection risk (67.5%), ensuring vaccine safety (56.7%) and the low risk of side effects (52.7%) were the most effective persuasions for promoting vaccinations.

**Conclusion:**

In China, older adolescents showed a higher prevalence for vaccine hesitancy than that of young adults. Abnormal illness behavior and history of physical diseases were risk factors for vaccine hesitancy among these older adolescents, while social support represents an important factor which could help to alleviate this hesitancy.

## Introduction

Since 2019, the COVID-19 pandemic has ravaged the world, with catastrophic effects on people's health and well-being (1). Fortunately, vaccinations have proved to be highly effective in reducing the morbidity and mortality of COVID-19 and offer the hope that mass vaccinations may end this pandemic (2, 3). However, results from recent worldwide surveys have revealed that merely 50–60% of respondents are willing to be vaccinated against COVID-19, with wide variations in these results across countries (4). Despite compelling evidence demonstrating the effectiveness of these vaccinations in saving millions of people from disease and disability, vaccine hesitancy remains a pervasive concern in recent years (5). In fact, COVID-19 vaccine hesitancy has become a global problem associated with the COVID-19 epidemic (6).

As defined by the World Health Organization (WHO) in March 2012, vaccine hesitancy refers to a refusal or delay in acceptance of a vaccination despite availability of vaccination services (5, 7). Vaccine hesitancy is a complex issue influenced by factors such as complacency, convenience and confidence (5). Recent findings on COVID-19 vaccine hesitancy have indicated that the main reasons for hesitancy include a lack of confidence in vaccine safety and efficacy (8–10). While social support from family, friends, colleagues and the community may be beneficial in promoting confidence in the safety and efficacy of the COVID-19 vaccine (11, 12), psychosocial factors such as psychological distress and abnormal illness behavior may undermine this confidence (13, 14). As a mass administration of this vaccine is critical for controlling the COVID-19 epidemic, an urgent need exists to identify the reasons for vaccine hesitancy and assess its possible risk factors.

In China, as well as throughout the world, COVID-19 vaccines have been preferentially developed and administered for use in adults vs. adolescents and children (15). Accordingly, current research on vaccine hesitancy in China has focused on adults over 18 years old (9, 16, 17). Conspicuously absent is any research on vaccine hesitancy in older adolescents and young adults. Vaccine hesitancy among older adolescents and young adults represents an area of particular concern for a number of reasons (18). First, older adolescents and young adults are more likely to be overly optimistic and complacent, and thus underestimate the existence of risks associated with this condition (9, 19, 20). It has been reported that this age group considers vaccination a low-priority, due to their optimistic judgment that no risk exists for their COVID-19 infection (21). In addition, the increased incidence of psychosocial problems during the COVID-19 epidemic, such as psychological distress experienced by older adolescents or young adults (22, 23), may undermine their confidence in COVID-19 vaccine safety and efficacy. Another major source of concern associated with this age group is that once infected, the epidemic may spread more rapidly and have severe consequences due to the frequency of their congregation in schools and/or places of social gatherings (24). Therefore, vaccine hesitancy in older adolescents and young adults is particularly troubling and requires investigation to enable the development of specific strategies that can be applied for the mitigation of this issue.

To the best of our knowledge, this study represents the first large-scale national study conducted on COVID-19 vaccine hesitancy among older adolescents and young adults in China. To address this issue, we assessed the prevalence and reasons for vaccine hesitancy, as well as the potential risk factors associated with this behavior.

## Materials and Methods

### Design, Subjects, and Procedure

This was a cross-sectional study conducted through an online survey over the period from March 14 to April 15, 2021. The study was performed 3 months after Chinese adults over 18 years old began receiving free COVID-19 vaccines, during which no vaccines were available for adolescents (25). COVID-19 vaccinations are now currently available for adolescents and children in China.

Older adolescents (16–17 years old) and young adults (18–21 years old) in China were encouraged to participate in an online survey via the applet with a questionnaire that was distributed on the WeChat platform. This online survey covered a wide range of questions, such as sociodemographics, clinical variables, willingness for vaccination, psychosocial indices as well as other related issues. A math question (86 – 7 = ?) was included to reduce the risk of irresponsible responses and provided an assurance for the quality of the questionnaire. Participants failing to complete the survey received a notice regarding unanswered questions when submitting their questionnaire. This study was approved by the Medical Ethics Committee of the Second Hospital of Shandong University [No. KYLL-2021(LW)-045].

### Measurements

#### Demographic Data

Age, sex, occupational classification (student vs. non-student), education level (≤9 years, i.e., junior high school and lower, vs. > 9 years, i.e., senior high school and higher), residence (urban vs. rural), yearly household income (≤30,000 RMB, 30,000–150,000 RMB, and ≥150,000 RMB) and self-reported health status (good vs. poor) were collected from the questions of the online survey. In addition, participants were also asked whether they had a history of physical diseases (“Do you have any organic disease diagnosed by medical examination in the hospital, such as hypertension, diabetes, heart disease or stroke, etc?”) and whether they had a history of mental illness (“Do you have any mental illness clearly diagnosed by a psychiatrist, such as schizophrenia, mania or depression, etc?”), whether they often consumed coffee or tea (i.e., drinking coffee or tea almost every day), whether they were current alcohol drinkers (i.e., drinking alcohol at least three times a week for more than 6 months) or current smokers (i.e., smoking almost every day for more than 6 months), whether they or their family members had been infected with COVID-19, whether they had received vaccinations in the past 3 years and whether they attended any lectures about COVID-19 vaccination.

#### COVID-19 Vaccine Hesitancy

**Figure 1:**
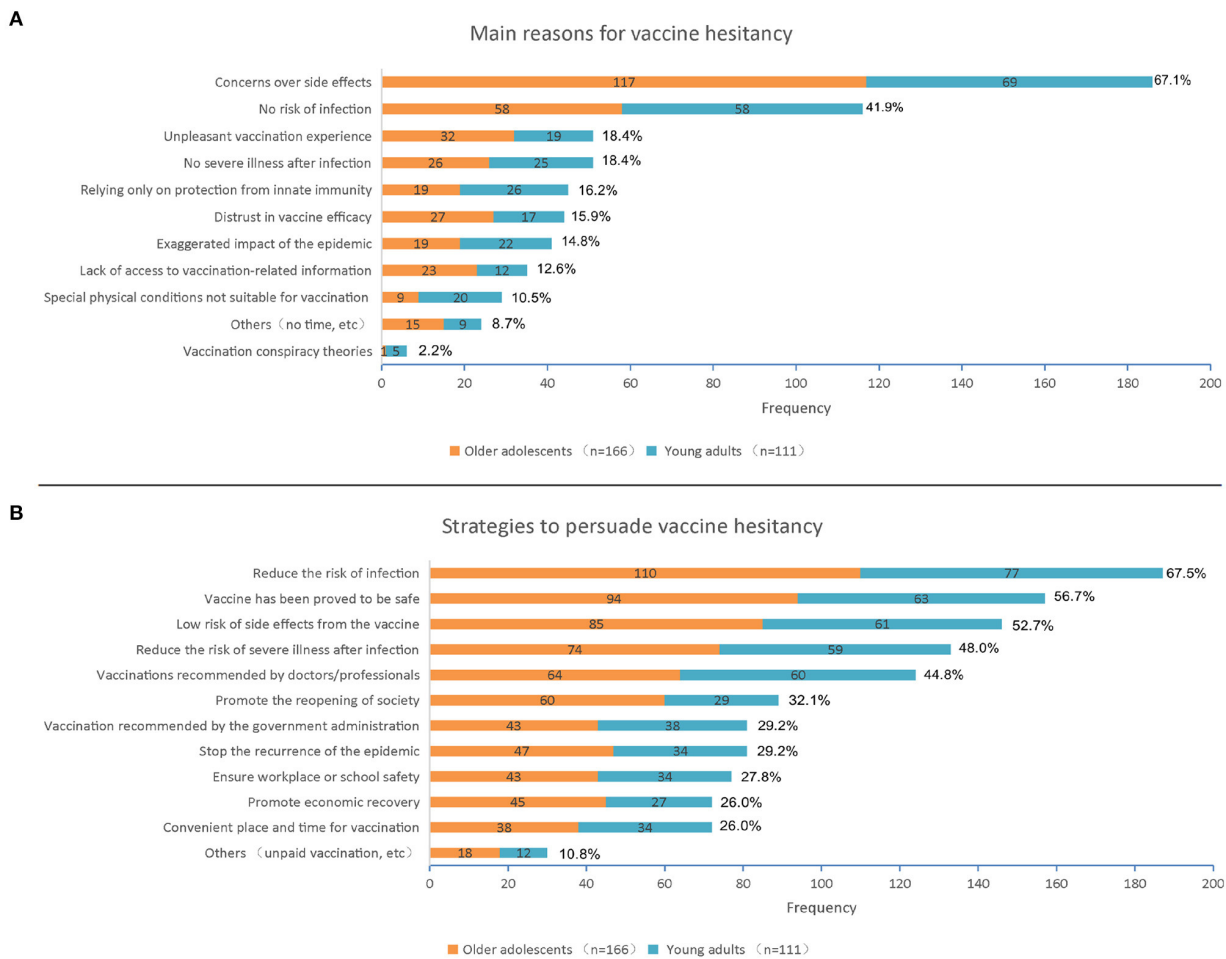
**(A)** Main reasons for COVID-19 vaccine hesitancy within older adolescents (*N* = 166) and young adults (*N* = 111); **(B)** Strategies to persuade COVID-19 vaccine hesitancy within older adolescents (*N* = 166) and young adults (*N* = 111). COVID-19–the coronavirus disease 2019.

Our primary assessment involved vaccine hesitancy, as determined by responses to the following question: “If a COVID-19 vaccine was available for you, would you like to get it?”. Those replying “no” or “uncertain” were identified as demonstrating vaccine hesitancy. These respondents were then asked: “What are the main reasons you would not take the vaccine?”, and were then directed to select at least one of the 11 possible answers as contained in Figure 1A. They were also asked: “Which strategies would increase your chances of getting a COVID-19 vaccine?”, and again were provided with a list of 12 possible options as shown in Figure 1B.

#### Factors From the Psychosocial Index-Young (PSI-Y)

The Psychosocial Index (PSI) is a self-rating scale based on clinimetric principles. It can be integrated by observer-rated clinical judgments and provides a first-line, comprehensive assessment of stress, well-being, distress, illness behavior and quality of life. A 51-item modified version of the PSI has been modified for assessing psychosocial factors among adolescents and young adults up to 21 years old (PSI-Young; PSI-Y), with particular reference to study activities, educational setting, peer relationships and family environment (26). This modified PSI-Y consisted of 11 items on sociodemographic and clinical variables with the remaining 40 items being contained in 5 domains: (1) stress (items 12–19 and 20–26 involving perceived and objective stress, life events and chronic stress; total scale scores ranging from 0 to 15), (2) well-being (items 27–32 involving positive relations with others, environmental mastery and autonomy; total scale scores ranging from 0 to 6), (3) psychological distress (items 33–47 consisting of a checklist of symptoms addressing sleep disturbances, somatization, anxiety, depression and irritability; total scale scores ranging from 0 to 45), (4) abnormal illness behavior (items 48–50 allowing the assessment of hypochondriacal beliefs and bodily preoccupations; total scale scores ranging from 0 to 9), and (5) quality of life (item 51 including a simple direct question on quality of life; total scale scores ranging from 0 to 4). Psychological well-being (0–6) and quality of life (0–4) scores could also be combined to generate a global well-being score (27). Abnormal illness behavior was defined by Pilowsky as: “the persistence of a maladaptive mode of experiencing, perceiving, evaluating, and responding to one's own health status, despite the fact that a doctor has provided a lucid and accurate appraisal of the situation and management to be followed (if any), with opportunities for discussion, negotiation and clarification, based on adequate assessment of all relevant biological, psychological, social, and cultural factors” (28). Here, its scale consists of 3 questions involving hypochondriacal beliefs and bodily preoccupations, with four possible choices ranging from absent abnormal illness behavior to maximum (i.e., not at all, only a little, somewhat, and a great deal). The attributable score to single items ranges from 0 to 3, and total score may vary from 0 to 9. Higher scores indicate a higher level of abnormal illness behavior (27). The PSI has been employed in series of clinical populations and has demonstrated a high degree of sensitivity as well as the capacity to significantly discriminate among varying degrees of psychosocial impairment.

#### Social Support Assessment

The Social Support Rating Scale (SSRS) is a 10-item self-report questionnaire used to assess the level of individual social support in the past year (29). It consists of three subscales: subjective support (items 1 and 3–5), objective support (items 2, 6, and 7) and utilization of support (items 8–10). Subjective support refers to the perceived social support people believe they receive from the support, care and help of family, friends, and colleagues. Objective support refers to visible, realistic, and direct support. Utilization of support incarnates the degree of application of social support. Higher scores for each subscale indicate a higher level of social support (29, 30).

#### Statistical Analyses

Data analyses were performed with use of the IBM SPSS Statistical Software program (version 23). A two-tailed *p* < 0.05 was required for results to be considered as statistically significant. For categorical variables, *p*-values were calculated using χ^2^ or Fisher exact tests. For continuous variables, *p*-values were calculated using the Wilcoxon rank-sum test. Subgroup analyses were performed for vaccine hesitancy and acceptance.

Vaccine hesitancy was used as a dependent variable, while independent variables included: individual demographics (i.e., age, sex, occupation, education level, residence, yearly household income), clinical variables (i.e., self-report of health, physical disease history, mental illness history, vaccination history in the last 3 years, their own or family COVID-19 infection history), lifestyle factors, attendance at COVID-19 lectures, PSI-Y domains (including stress, psychological distress, abnormal illness behavior, global well-being), and SSRS determinations (i.e., objective, subjective and/or utilization of support). Multivariate logistic regression analyses were conducted using stepwise variable selection with different variables entered into the models to assess independent influence for vaccine hesitancy among older adolescents and young adults. The variables in Model 1 included variables of individual demographics, clinical variables, lifestyle factors, and attendance at COVID-19 lectures, then the variables in Model 2 additionally included PSI-Y domains and SSRS determinations, that is, all independent variables.

## Results

A total of 2,429 participants throughout China completed the survey. As 15 participants (6.18/‰) incorrectly answered the math question they were excluded leaving 2,414 subjects for final analyses.

Sociodemographic characteristics of the older adolescents (*N* = 1,009) vs. young adults (*N* = 1,405) are summarized in Table 1. Compared with young adults, older adolescents showed higher prevalence rates of COVID-19 vaccine hesitancy (16.5 vs. 7.9%, *p* < 0.001). Young adults had higher rates of vaccination history in the past 3 years (*p* < 0.001). Current smokers (*p* < 0.001) and alcohol drinkers (*p* = 0.003) were more common among young adults (*p* = 0.003), while current coffee or tea drinkers accounted for a higher proportion of older adolescents (*p* < 0.001). Young adults showed higher rates of attendance at COVID-19 lectures (*p* < 0.001). Older adolescents had higher scores for psychological distress (*p* < 0.001) and abnormal illness behavior (*p* < 0.001) than young adults. Older adolescents had higher total SSRS scores (*p* < 0.001), in particular for objective support (*p* < 0.001). Statistically significant differences between older adolescents and young adults were also found for sex (*p* < 0.001) and yearly household income (*p* < 0.001).

**Table 1:** Sociodemographic characteristics in older adolescents vs. young adults.

**Characteristics**	**Total**	**Older adolescents**	**Young adults**	***p-*Value**
	**(*n* = 2,414)**	**(*n* = 1,009)**	**(*n* = 1,405)**	
Vaccine hesitancy	11.5 (277)	16.5 (166)	7.9 (111)	<0.001
Age, years	18.03 ± 1.55	16.46 ± 0.63	19.16 ± 0.88	<0.001
Male sex	40.3 (973)	47.0 (474)	35.5 (499)	<0.001
Educational level >9 years	99.0 (2,390)	98.7 (996)	99.2 (1,394)	0.217
Student	98.6 (2,381)	98.6 (995)	98.6 (1,386)	0.941
Residence				0.122
Urban	36.4 (878)	38.2 (385)	35.1 (493)	
Rural	63.6 (1,536)	61.8 (624)	64.9 (912)	
Household income per year, RMB				<0.001
≤ 30,000	33.8 (815)	25.4 (256)	39.8 (559)	
>30,000 and <150,000	56.7 (1,369)	61.8 (624)	53.0 (745)	
≥150,000	9.5 (230)	12.8 (129)	7.2 (101)	
Good self-reported health status	69.4 (1,675)	69.7 (703)	69.2 (972)	0.796
History of physical diseases	4.3 (105)	4.3 (43)	4.4 (62)	0.857
History of mental illness	1.7 (41)	1.4 (14)	1.9 (27)	0.316
**Lifestyle factors**				
Current coffee or tea drinkers	55.2 (1,332)	61.2 (618)	50.8 (714)	<0.001
Current smokers	5.3 (127)	3.4 (34)	6.6 (93)	<0.001
Current alcohol drinkers	6.5 (157)	4.8 (48)	7.8 (109)	0.003
Vaccination history in the past 3 years	46.4 (1,121)	33.8 (341)	55.5 (780)	<0.001
COVID-19 infection history	0.4 (10)	0.4 (4)	0.4 (6)	0.908*
COVID-19 infection in family members	0.5 (12)	0.6 (6)	0.4 (6)	0.564
Attendance of COVID-19 lecture	48.2 (1,164)	41.8 (422)	52.8 (742)	<0.001
**Factors from the PSI-Y**				
Stress	3.15 ± 2.30	3.15 ± 2.29	3.14 ± 2.31	0.805
Global well-being	6.90 ± 1.86	6.84 ± 1.88	6.94 ± 1.84	0.166
Psychological distress	11.89 ± 8.66	13.27 ± 8.82	10.90 ± 8.41	<0.001
Abnormal illness behavior	1.67 ± 1.83	1.96 ± 1.85	1.47 ± 1.79	<0.001
**SSRS**				
Objective support	7.21 ± 2.77	7.84 ± 2.60	6.76 ± 2.80	<0.001
Subjective support	20.71 ± 4.21	20.87 ± 4.26	20.59 ± 4.18	0.219
Utilization of support	7.66 ± 2.14	7.66 ± 2.13	7.66 ± 2.15	0.931
Total score	35.57 ± 7.15	36.36 ± 7.01	35.01 ± 7.19	<0.001

Values are presented as means ± SD or percents (n). For categorical variables, p-values were calculated using χ2 or Fisher exact tests, and ^*^p-values are obtained from Fisher exact tests; for continuous variables, p-values were calculated using the Wilcoxon rank-sum test. COVID-19, the coronavirus disease 2019; PSI-Y, Psychosocial Index-Young; SSRS, Social Support Rating Scale.

Table 2 contains a summary of the descriptive characteristics and comparisons of the vaccine acceptance vs. hesitancy groups among older adolescents and young adults. Among older adolescents, the vaccine hesitancy group reported a poorer health status (*p* < 0.001) and higher rates of physical disease history (*p* = 0.013) than that of the vaccine acceptance group. The vaccine hesitancy group had higher scores for stress (*p* = 0.009), psychological distress (*p* = 0.001) and abnormal illness behavior (*p* < 0.001), while the vaccine acceptance group had higher total scores for social support (*p* < 0.001), of which scores for objective support (*p* = 0.001), subjective support (*p* = 0.003) and utilization of support (*p* < 0.001) were all higher than that of the vaccine hesitancy group. Among young adults, current smokers (*p* = 0.002) and alcohol drinkers (*p* = 0.018) were more common within the vaccine hesitancy vs. acceptance group. The vaccine acceptance group showed higher scores for global well-being (*p* < 0.001), objective support (*p* < 0.001), subjective support (p <0.001) and utilization of support (*p* < 0.001) than that of the vaccine hesitancy group.

**Table 2:** General characteristics by vaccine hesitancy classification among older adolescents and young adults.

**Characteristics**	**Older adolescents (*****n*** **= 1,009)**		***p-*Value**	**Young adults (*****n*** **= 1,405)**		***p-*Value**
	**Acceptance**	**Hesitancy**		**Acceptance**	**Hesitancy**	
Subjects	83.5 (843)	16.5 (166)		92.1 (1,294)	7.9 (111)	
Age, years	16.47 ± 0.62	16.43 ± 0.68	0.734	19.17 ± 0.88	19.05 ± 0.92	0.149
Male sex	47.6 (401)	44.0 (73)	0.397	35.5 (460)	35.1 (39)	0.930
Educational level >9 years	98.5 (830)	100.0 (166)	0.143*	99.3 (1,285)	98.2 (109)	0.214*
Student	98.7 (832)	98.2 (163)	0.713*	98.7 (1,277)	98.2 (109)	0.658*
Residence			0.683			0.992
Urban	38.4 (324)	36.7 (61)		35.1 (454)	35.1 (39)	
Rural	61.6 (519)	63.3 (105)		64.9 (840)	64.9 (72)	
Household income per year, RMB			0.699			0.459
≤ 30,000	24.9 (210)	27.7 (46)		39.9 (516)	38.7 (43)	
>30,000 and <150,000	62.4 (526)	59.0 (98)		52.7 (682)	56.8 (63)	
≥150,000	12.7 (107)	13.3 (22)		7.4 (96)	4.5 (5)	
Good self-reported health status	81.9 (690)	68.1 (113)	<0.001	68.6 (888)	75.7 (84)	0.123
History of physical diseases	3.6 (30)	7.8 (13)	0.013	4.2 (54)	7.2 (8)	0.145*
History of mental illness	1.7 (14)	0.0 (0)	0.144*	1.8 (23)	3.6 (4)	0.159*
**Lifestyle factors**						
Current coffee or tea drinkers	60.9 (513)	63.3 (105)	0.562	50.0 (647)	57.7 (64)	0.121
Current smokers	3.1 (26)	4.8 (8)	0.257	6.0 (78)	13.5 (15)	0.002
Current alcohol drinkers	4.9 (41)	4.2 (7)	0.720	7.3 (94)	13.5 (15)	0.018
Vaccination history in the past 3 years	33.6 (283)	34.9 (58)	0.733	55.1 (713)	60.4 (67)	0.285
COVID-19 infection history	0.4 (3)	0.6 (1)	0.513*	0.4 (5)	0.9 (1)	0.390*
COVID-19 infection in family members	0.6 (5)	0.6 (1)	1.000*	0.4 (5)	0.9 (1)	0.390*
Attendance of COVID-19 lecture	42.1 (355)	40.4 (67)	0.676	53.0 (686)	50.5 (56)	0.604
**Factors from the PSI-Y**						
Stress	3.06 ± 2.23	3.63 ± 2.50	0.009	3.11 ± 2.28	3.50 ± 2.57	0.164
Global well-being	6.90 ± 1.85	6.56 ± 2.02	0.063	7.00 ± 1.81	6.15 ± 1.96	<0.001
Psychological distress	12.81 ± 8.59	15.57 ± 9.60	0.001	10.84 ± 8.33	11.61 ± 9.34	0.640
Abnormal illness behavior	1.86 ± 1.82	2.45 ± 1.91	<0.001	1.44 ± 1.76	1.82 ± 2.09	0.102
**SSRS**						
Objective support	7.95 ± 2.59	7.28 ± 2.58	0.001	6.87 ± 2.76	5.51 ± 2.98	<0.001
Subjective support	21.05 ± 4.23	19.98 ± 4.32	0.003	20.74 ± 4.14	18.86 ± 4.23	<0.001
Utilization of support	7.77 ± 2.10	7.07 ± 2.22	<0.001	7.77 ± 2.11	6.37 ± 2.12	<0.001
Total score	36.77 ± 6.90	34.33 ± 7.26	<0.001	35.38 ± 7.06	30.74 ± 7.23	<0.001

Values are presented as means ± SD or percents (n). For categorical variables, p-values are calculated using χ2 or Fisher exact tests, and ^*^p-values are obtained from Fisher exact tests; for continuous variables, p-values are calculated using the Wilcoxon rank-sum test. COVID-19, the coronavirus disease 2019; PSI-Y, Psychosocial Index-Young; SSRS, Social Support Rating Scale.

Results from the multivariate logistic regression analyses (Table 3) indicated that history of physical diseases [odds ratio (OR), 2.58, 95% confidence interval (CI), 1.30–5.15; *p* = 0.007] and abnormal illness behavior (OR, 1.17; 95% CI, 1.07–1.28; *p* = 0.001) were risk factors associated with vaccine hesitancy among older adolescents, while only a good self-reported health status (OR, 1.66; 95% CI, 1.00–2.74; *p* = 0.048) was a risk factor for young adults. In contrast, within older adolescents, utilization of support (OR, 0.86; 95% CI, 0.79–0.94; *p* = 0.001) was the only protective factor associated with a resistance for vaccine hesitancy, while in young adults two protective factors were identified, global well-being (OR, 0.86; 95% CI, 0.77–0.97; *p* = 0.010) and utilization of support (OR, 0.77; 95% CI, 0.68–0.86; *p* < 0.001).

**Table 3:** Multivariate conditional logistic regression for vaccine hesitancy risk among older adolescents and young adults.

**Variables**	**Model 1**			**Model 2**		
	**OR (95%CI)**	***p*-Value**	**R^**2**^**	**OR (95%CI)**	***p*-Value**	**R^**2**^**
**Older adolescents**			0.036			0.097
Abnormal illness behavior				1.17 (1.07, 1.28)	0.001	
Utilization of support				0.86 (0.79, 0.94)	0.001	
History of physical diseases	2.46 (1.19, 5.08)	0.015		2.58 (1.30, 5.15)	0.007	
**Young adults**			0.062			0.114
Global well-being				0.86 (0.77, 0.97)	0.010	
Utilization of support				0.77 (0.68, 0.86)	<0.001	
Good self-reported health status	1.73 (1.06, 2.81)	0.027		1.66 (1.00, 2.74)	0.048	
Current coffee or tea drinkers	2.29 (1.15, 4.57)	0.019		1.69 (0.83, 3.44)	0.147	
**Total population**			0.045			0.101
Abnormal illness behavior				1.10 (1.03, 1.17)	0.005	
Subjective support				0.92 (0.87, 0.97)	0.002	
Utilization of support				0.83 (0.78, 0.89)	<0.001	
History of physical diseases	2.04 (1.23, 3.37)	0.006		2.29 (1.36, 3.84)	0.002	
Current smokers	2.16 (1.34, 3.50)	0.002		1.79 (1.09, 2.94)	0.023	
Age, years	0.75 (0.69, 0.82)	<0.001		0.74 (0.67, 0.80)	<0.001	

*OR, odds ratio; CI, confidence interval. Multivariate logistic regression analyses using stepwise variable selection. Independent variables in Model 1 and 2 are as follows: Model 1: individual demographics (i.e., age, sex, occupation, education level, residence, yearly household income), clinical variables (i.e., self-report of health, physical disease history, mental illness history, vaccination history in the last 3 years, their own or family COVID-19 infection history), lifestyle factors and attendance at COVID-19 lectures; and Model 2: individual demographics, clinical variables, lifestyle factors, attendance at COVID-19 lectures, PSI-Y domains (i.e., stress, psychological distress, abnormal illness behavior, global well-being) and SSRS determinations (i.e., objective, subjective and utilization of support). COVID-19, the coronavirus disease 2019; PSI-Y, Psychosocial Index-Young; SSRS, Social Support Rating Scale*.

Figure 1A contains a summary of the main reasons for vaccine hesitancy as obtained from 166 older adolescents and 111 young adults. From these 277 subjects, concerns over side effects of a COVID-19 vaccine (*n* = 186; 67.1%) and thinking they have no risk for infection (*n* = 116; 41.9%) were the most prevalent reasons, followed by thinking that there would be no severe illness after infection and an unpleasant effect of the vaccination would be experienced (*n* = 51; 18.4%). With regard to differences in the main reasons for vaccine hesitancy between older adolescents vs. young adults, thinking that there was no risk for infection (*p* = 0.004), relying only on protection from innate immunity (*p* = 0.008), special physical conditions not suitable for vaccination (*p* = 0.001) and vaccination conspiracy theories (*p* = 0.040) were more common among young adults than older adolescents (Supplementary Table 1). As shown in Figure 1B, when asked which strategies would increase their chances of getting a COVID-19 vaccine, 67.5% of total vaccine hesitators reported that they would if the vaccine could reduce the risk of COVID-19 infection, 56.7% would be persuaded to get a vaccine after the vaccine had been proved to be safe, and 52.7% if there was a low risk for side effects from the vaccine. When comparing the two groups, young adults were found to be more likely to believe and accept the advice on vaccination as recommended by doctors or professionals than older adolescents (*p* = 0.011) (Supplementary Table 2).

## Discussion

A number of novel findings have emerged from this first large-scale nationwide study on the willingness for COVID-19 vaccination and causes for vaccine hesitancy among older adolescents and young adults in China. First, older adolescents showed higher prevalence rates of vaccine hesitancy as compared with young adults. Second, a history of physical diseases and abnormal illness behavior were risk factors for vaccine hesitancy in older adolescents while a good self-reported health status was a risk factor for young adults. Third, the main reasons for vaccine hesitancy consisted of concerns regarding side effects of the COVID-19 vaccine and their belief that they had no risk for infection. To mitigate these vaccination hesitancy concepts, evidence indicating a reduction in the risk of infection from vaccines, assurances of its safety and providing information on the low risk of side effects were the most effective arguments. Therefore, these findings provide the first evidence for development of a public health strategy to alleviate vaccine hesitancy among older adolescents and young adults, which may then contribute to a wide-spread increase in COVID-19 vaccination.

Vaccine hesitancy, which is listed as one of the top 10 threats by the WHO in 2019, has become a problem of worldwide concern in recent years (7, 31). This issue has become particularly challenging with the increasing need for vaccinations against COVID-19. As of May 2021, nearly 1.9 billion doses of COVID-19 vaccines had been administered worldwide (32). According to clinical reports, this COVID-19 vaccination is very effective in reducing severe illness and deaths from COVID-19, and the vaccine has been shown to be safe with extremely low risks of side effects (15, 33, 34). However, COVID-19 vaccine hesitancy remains a common problem worldwide, and can derail efforts to end the current pandemic (35–37). Current research on COVID-19 vaccine hesitancy in China has focused on adults (9, 38), while an evaluation of this issue within older adolescents and young adults has been conspicuously absent. Therefore, this study was performed as a means to correct this deficit. Our findings revealed that the incidence of COVID-19 vaccine hesitancy among older adolescents was ~16.5%, which was greater than that in young adults. These results indicate that the emphasis in promoting COVID-19 vaccinations should be directed toward older adolescents.

The potential risk factors for older adolescents and young adults, as identified in our study, are related to issues such as complacency, convenience and confidence, all of which contribute to the development of vaccine hesitancy (5). We found that a history of physical diseases and abnormal illness behavior were risk factors for vaccine hesitancy among older adolescents. Very often, when people with a history of physical diseases consider COVID-19 vaccination, their first concern is whether their pre-existing diseases will worsen or recur as a result of the vaccination. In particular, older adolescents, who are prone to experience serious neurological or immune-related diseases, such as asthma, nephritis and epilepsy, would have their initial vaccine hesitancy assuaged only when provided with clear evidence of low COVID-19 vaccine risk from medical experts. On the other side, individuals with abnormal disease behaviors demonstrate inappropriate responses in their ability to evaluate and act upon their symptoms (39). Such individuals tend to exhibit hypochondriacal beliefs and excessive physical concerns when seeking professional help, which could then affect the formation of a trusting relationship (40). The presence of abnormal illness behavior among older adolescents affects their confidence in COVID-19 vaccine safety and efficacy, despite professional certification. As a result, vaccine hesitancy remains. Interestingly, to our knowledge, abnormal illness behavior has never been reported or studied as a risk factor for vaccine hesitancy. We also found that a good self-reported health status was a risk factor for vaccine hesitancy in young adults, as it seems that these individuals appear to be more complacent about their health status and therefore ignore the importance of vaccination. Predictably, when people resist their COVID-19 vaccination and rely solely on their own innate immunity they produce an extremely hazardous condition for themselves as well as others.

When investigating the main reasons for COVID-19 vaccine hesitancy among older adolescents and young adults, we found that concerns over side effects of the COVID-19 vaccine were the most common reason, which is consistent with that of previous findings as obtained with Chinese adults (8, 9, 41). Fortunately, once statistics on the safety of the COVID-19 vaccine and low risk of side effects have been presented, more than half of the hesitators in our study were persuaded to be vaccinated. However, beliefs that there were no risks of infection or no severe illness after infection with COVID-19 remained, and we further observed that beliefs of invulnerability regarding infection risk as well as relying only on protection from innate immunity were more common among young adults than older adolescents. As previously reported, young adults are more likely to be overly optimistic and complacent, leading to an underestimation of risk hazards (19, 20). However, a current news report on the rapid resurgence of the epidemic in Guangzhou, China (42) appears to refute this “optimistic bias.” These differences between older adolescents and young adults highlight the need for the identification of specific considerations and programs when developing effective strategies for addressing vaccine hesitancy within different subgroups (43).

Given the complexity of factors contributing to COVID-19 vaccine hesitancy, no single intervention can effectively address this issue in its entirety (17, 37, 44, 45). Fortifying information regarding the importance, safety and efficacy of COVID-19 vaccines among older adolescents and young adults is a worthwhile endeavor. In this regard, online videos or applets from officials or professionals may be helpful (46) along with providing access to appropriate psychological interventions especially for older adolescents who exhibit vaccination hesitancy to vaccines (41, 47). Such procedures may help dispel concerns about side effects of the COVID-19 vaccine. In addition, increased social support from family members, friends, professional medical workers and public health departments, would exert a positive effect on reducing vaccine hesitancy and promoting acceptance of the vaccine (12, 48).

### Limitations

This study has some limitations requiring consideration. First, given the unknown characteristics of those who did not participate in the survey, the issue of whether the data obtained is representative of older adolescents and young adults within the general Chinese population cannot be determined. Second, as the psychological evaluations were based on online surveys consisting of self-reports, these responses may not be entirely reliable. Therefore, use of clinical interviews will be needed to obtain a more comprehensive and objective assessment. Third, as the data were collected 1 year ago, prior to the promotion of a COVID-19 vaccine booster dose, our results may not reflect the current level of vaccine hesitancy in older Chinese adolescents and young adults. Finally, the voluntary nature of this survey makes it difficult to perform sufficient follow-up assessments of the participants.

## Conclusion

In China, a higher prevalence of COVID-19 vaccine hesitancy was found among older adolescents as compared with young adults, with a history of physical disease and abnormal illness behavior being risk factors for this hesitancy. The most common reason for vaccine hesitancy was a concern over side effects of the COVID-19 vaccine. A number of approaches are suggested to address vaccine hesitancy, including providing increased information regarding the importance, safety and efficacy of the vaccine and access to social support networks.

## Data Availability Statement

The original contributions presented in the study are included in the article/Supplementary Material, further inquiries can be directed to the corresponding authors.

## Ethics Statement

The studies involving human participants were reviewed and approved by the Medical Ethics Committee of the Second Hospital of Shandong University [No. KYLL-2021(LW)-045]. Written informed consent to participate in this study was provided by the participants' legal guardian/next of kin.

## Author Contributions

XZ: conception and design, drafting of the manuscript, and critical revision of the manuscript for important intellectual content. PZ, YaL, HuaW, PW, HuiW, ZM, QL, ShiZ, SheZ, NL, JZ, HY, YuL, and JB: conduction. XZ and LL: statistical analysis. PZ, HuaW, PW, ZM, QL, JZ, and YaL: administrative, technical, or material support. All authors read and approved the final paper.

## Funding

This study was supported by grants from the Rongxiang Regenerative Medicine Foundation of Shandong University (No. 2019SDRX-09).

## Conflict of Interest

The authors declare that the research was conducted in the absence of any commercial or financial relationships that could be construed as a potential conflict of interest.

## Publisher's Note

All claims expressed in this article are solely those of the authors and do not necessarily represent those of their affiliated organizations, or those of the publisher, the editors and the reviewers. Any product that may be evaluated in this article, or claim that may be made by its manufacturer, is not guaranteed or endorsed by the publisher.
