# Supplementary material for: COVID-19 Vaccine Hesitancy Among Older Adolescents and Young Adults: A National Cross-Sectional Study in China
Source: Front Public Health. 2022 May 12;10:877668. doi: 10.3389/fpubh.2022.877668 (PMC9133905; doi:10.3389/fpubh.2022.877668)
Supplement: Supplementary file 1 [file Data_Sheet_1.docx]

**Supplementary Table 1.** Main reasons for COVID-19 vaccine hesitancy among older adolescents versus young adults.

| Main reasons for vaccine hesitancy | Total | Older adolescents | Young adults | *p* value |
| --- | --- | --- | --- | --- |
|  | (n=277) | (n= 166) | (n= 111) |  |
| Concerns over side effects | 67.1 (186) | 70.5 (117) | 62.2 (69) | 0.149 |
| No risk of infection | 41.9 (116) | 34.9 (58) | 52.3 (58) | 0.004 |
| No severe illness after infection | 18.4 (51) | 15.7 (26) | 22.5 (25) | 0.149 |
| Relying only on protection from innate immunity | 16.2 (45) | 11.4 (19) | 23.4 (26) | 0.008 |
| Exaggerated impact of the epidemic | 14.8 (41) | 11.4 (19) | 19.8 (22) | 0.054 |
| Special physical conditions not suitable for vaccination | 10.5 (29) | 5.4 (9) | 18.0 (20) | 0.001 |
| Unpleasant vaccination experience | 18.4 (51) | 19.3 (32) | 17.1 (19) | 0.649 |
| Distrust in vaccine efficacy | 15.9 (44) | 16.3 (27) | 15.3 (17) | 0.832 |
| Lack of access to vaccination-related information | 12.6 (35) | 13.9 (23) | 10.8 (12) | 0.455 |
| Others(no time, etc) | 8.7 (24) | 9.0 (15) | 8.1 (9) | 0.788 |
| Vaccination conspiracy theories | 2.2 (6) | 0.1 (1) | 4.5 (5) | 0.040 |
| Values are presented as percents (n). χ^2^ tests or Fisher exact tests for group differences of categorical variables. COVID-19, the coronavirus disease 2019. | | | | |

**Supplementary Table 2.** Strategies to persuade COVID-19 vaccine hesitancy among older adolescents versus young adults.

| Strategies to persuade vaccine hesitancy | Total | Older adolescents | Young adults | *p* value |
| --- | --- | --- | --- | --- |
|  | (n=277) | (n= 166) | (n= 111) |  |
| Reduce the risk of infection | 67.5 (187) | 66.3 (110) | 69.4 (77) | 0.589 |
| Vaccine has been proved to be safe | 56.7 (157) | 56.6 (94) | 56.8 (63) | 0.983 |
| Low risk of side effects from the vaccine | 52.7 (146) | 51.2 (85) | 55.0 (61) | 0.540 |
| Reduce the risk of severe illness after infection | 48.0 (133) | 44.6 (74) | 53.2 (59) | 0.162 |
| Vaccinations recommended by doctors/professionals | 44.8 (124) | 38.6 (64) | 54.1 (60) | 0.011 |
| Convenient place and time for vaccination | 26.0 (72) | 22.9 (38) | 30.6 (34) | 0.150 |
| Promote the reopening of society | 32.1 (89) | 36.1 (60) | 26.1 (29) | 0.080 |
| Stop the recurrence of the epidemic | 29.2 (81) | 28.3 (47) | 30.6 (34) | 0.678 |
| Ensure workplace or school safety | 27.8 (77) | 25.9 (43) | 30.6 (34) | 0.389 |
| Promote economic recovery | 26.0 (72) | 27.1 (45) | 24.3 (27) | 0.605 |
| Vaccination recommended by the government administration | 29.2 (81) | 25.9 (43) | 34.2 (38) | 0.135 |
| Others (unpaid vaccination, etc) | 10.8 (30) | 10.8 (18) | 10.8 (12) | 0.993 |
| Values are presented as percents (n). χ^2^ tests for group differences of categorical variables. COVID-19, the coronavirus disease 2019. | | | | |
